# Risk – benefit perception and consumption of seafood in European consumers

**DOI:** 10.1186/2049-3258-72-S1-P8

**Published:** 2014-06-06

**Authors:** Silke Jacobs, Isabelle Sioen, Stefaan De Henauw, Núria Tous, Ana Luísa Maulvault, Gabriella Fait, Federico Cardona Pons, Wim Verbeke

**Affiliations:** 1 Department of Agricultural Economics, Ghent University, B-9000 Ghent, Belgium; 2 Department of Public health, Ghent University, B-9000 Ghent, Belgium; 3 Laboratory of Toxicology and Environmental Health –Tecnatox, Universitat Rovira i Virgili (URV) School of Medicine, IISPV, 43201 Reus, Spain; 4 Division of Aquaculture and Upgrading (DivAV), Portuguese Institute for the Sea and Atmosphere (IPMA), 1449-006 Lisbon, Portugal; 5 Aeiforia Srl, 29027 Gariga di Podenzano (PC), Italy; 6 AquaTT, Dublin 2, Ireland

## 

Seafood consumption entails important potential health benefits such as lowering the risk of cardiovascular diseases. However, seafood may also be a source of environmental contaminants for which no maximum limits are set by authorities yet (*i.e.* priority contaminants). Exposure to these contaminants could imply health risks, especially for the more vulnerable consumer groups, such as pregnant women and children. Because of recent media attention to these contaminants, revealing the benefit and risk perception of European consumers toward seafood is of particular interest.

For this purpose, a web based survey was performed in 2013 in five European countries, namely Belgium, Ireland, Italy, Spain, and Portugal (n=2917; age 18 to 75 years; 1451 women and 1466 men). Risk and benefit perception statements were scored on a 7-point Likert scale ranging from totally disagree to totally agree, forming a construct of seven and three items, respectively. Furthermore, consumers’ concern about seafood safety was also measured on a 7-point Likert scale.

The perceived benefits of consuming seafood outweigh the perceived risks among European consumers. In general, the mean score amounts 5.43 (± 1.34) for the benefit-construct versus 2.75 (± 1.50) for the risk-construct. But, importantly, a certain concern about seafood safety has to be underlined. More in particular, 42% of the participants are concerned about the safety of seafood and 39% are concerned about the amount of environmental contaminants in seafood. It should also be noted that Portugal has the highest seafood consumption and Belgium the lowest seafood consumption with a mean self-reported consumption of 487 and 191 gram per week, respectively. Interestingly, the Portuguese consumers indicated the lowest mean score on risk perception and the highest mean score on benefit perception, both significantly different from the mean scores of the other four countries. In addition, a weak negative association is measured between risk perception and consumption (r=-0.145, p<0.001) and a weak positive association is measured between benefit perception and consumption (r=0.214, p<0.001).

Because of a potential link between risk-benefit perception and seafood consumption, it is of great interest to determine the relationship between the risk-benefit perception and consumption patterns in further analyses.

